# The effect of neoadjuvant chemotherapy and chemoradiotherapy on exercise capacity and outcome following upper gastrointestinal cancer surgery: an observational cohort study

**DOI:** 10.1186/s12885-016-2682-6

**Published:** 2016-09-02

**Authors:** M. A. West, L. Loughney, G. Ambler, B. D. Dimitrov, J. J. Kelly, M. G. Mythen, R. Sturgess, P. M. A. Calverley, A. Kendrick, M. P. W. Grocott, S. Jack

**Affiliations:** 1 Anaesthesia and Critical Care Research Area, NIHR Respiratory Biomedical Research Unit, University Hospital Southampton NHS Foundation Trust, CE93 MP24, Tremona Road, Southampton, SO16 6YD UK; 2 Integrative Physiology and Critical Illness Group, Clinical and Experimental Sciences, Faculty of Medicine, University of Southampton, Tremona Road, Southampton, UK; 3 Academic Unit of Cancer Sciences, Faculty of Medicine, University of Southampton, Southampton, UK; 4 Department of Statistical Science, University College London, London, UK; 5 Academic Unit of Primary Care and Population Sciences, Faculty of Medicine, University of Southampton, Tremona Road, Southampton, UK; 6 Department of Surgery, University Hospital Southampton NHS Foundation Trust, Tremona Road, Southampton, UK; 7 Centre for Anaesthesia, Institute of Sport Exercise and Health, University College London Hospitals NIHR Biomedical Research Centre, London, UK; 8 Department of Gastroenterology, University Hospitals Aintree, Longmoor Road, Liverpool, UK; 9 Department of Respiratory Research, University of Liverpool, University Hospitals Aintree, Longmoor Road, Liverpool, UK; 10 Department of Physiological Sciences, University of Bristol, Bristol, UK

**Keywords:** Neoadjuvant, Chemotherapy, Chemoradiotherapy, Cancer, Cardiopulmonary, Exercise test, Fitness, Surgery, Outcome, Morbidity, Mortality

## Abstract

**Background:**

In 2014 approximately 21,200 patients were diagnosed with oesophageal and gastric cancer in England and Wales, of whom 37 % underwent planned curative treatments. Potentially curative surgical resection is associated with significant morbidity and mortality. For operable locally advanced disease, neoadjuvant chemotherapy (NAC) improves survival over surgery alone. However, NAC carries the risk of toxicity and is associated with a decrease in physical fitness, which may in turn influence subsequent clinical outcome. Lower levels of physical fitness are associated with worse outcome following major surgery in general and Upper Gastrointestinal Surgery (UGI) surgery in particular. Cardiopulmonary exercise testing (CPET) provides an objective assessment of physical fitness. The aim of this study is to test the hypothesis that NAC prior to upper gastrointestinal cancer surgery is associated with a decrease in physical fitness and that the magnitude of the change in physical fitness will predict mortality 1 year following surgery.

**Methods:**

This study is a multi-centre, prospective, blinded, observational cohort study of participants with oesophageal and gastric cancer scheduled for neoadjuvant cancer treatment (chemo- and chemoradiotherapy) and surgery. The primary endpoints are physical fitness (oxygen uptake at lactate threshold measured using CPET) and 1-year mortality following surgery; secondary endpoints include post-operative morbidity (Post-Operative Morbidity Survey (POMS)) 5 days after surgery and patient related quality of life (EQ-5D-5 L).

**Discussion:**

The principal benefits of this study, if the underlying hypothesis is correct, will be to facilitate better selection of treatments (e.g. NAC, Surgery) in patients with oesophageal or gastric cancer. It may also be possible to develop new treatments to reduce the effects of neoadjuvant cancer treatment on physical fitness. These results will contribute to the design of a large, multi-centre trial to determine whether an in-hospital exercise-training programme that increases physical fitness leads to improved overall survival.

**Trial registration:**

ClinicalTrials.gov NCT01325883 - 29^th^ March 2011.

## Background

Worldwide, oesophageal cancer is the eighth most common cancer and the sixth most common cause of cancer death, while gastric cancer is the fifth most common cancer and third most common cause of cancer-death. In England and Wales, approximately 21,200 patients were diagnosed with oesophageal or gastric cancer in 2014, of which 37 % underwent planned curative treatment [1, 2]. Although potentially curative, surgical resection is attempted in up to 80 % of those patients planned for curative treatments, however significant morbidity and mortality is reported. The reported 90-day mortality rates for oesophagectomy and gastrectomy are 4.4 % and 4.3 % respectively, with 1-year survival rates between 76.1 % and 78.0 % depending on the site of the primary tumour [1]. A large updated meta-analysis provides evidence that for operable OG disease neoadjuvant therapies improve survival over surgery alone [3]. In the UK the MAGIC trial has resulted in a practice change in favour of neoadjuvant chemotherapy (NAC) [4]. Treatment with neoadjuvant therapies carries the risk of toxicity and in clinical practice this may be associated with an increased risk of surgical morbidity [3]. Cardiopulmonary exercise testing (CPET) provides an objective assessment of physical fitness through evaluating cardio-respiratory function under the stress of exercise mimicking the stress of major surgery. Variables derived from CPET such as oxygen uptake at estimated lactate threshold ($\overset{.}{\mathrm{V}}{\mathrm{o}}_2$ at ${\widehat{\uptheta}}_{\mathrm{L}}$) and at peak exercise ($\overset{.}{\mathrm{V}}{\mathrm{o}}_2$ peak) are associated with worse outcome following UGI surgery [5, 6].

In a preliminary study, we showed, in a small number of patients, that neoadjuvant chemotherapy (NAC) before upper gastrointestinal (UGI) cancer surgery significantly reduced physical fitness [7]. In this study, lower baseline fitness was associated with reduced 1-year-survival in patients completing NAC and surgery, but not in patients who did not complete NAC. We therefore speculated that in some patients the harms of NAC may outweigh the benefits and set out to test the hypothesis that neo-adjuvant chemotherapy (or chemoradiotherapy) was associated with reduced physical fitness ($\overset{.}{\mathrm{V}}{\mathrm{o}}_2$ at ${\widehat{\uptheta}}_{\mathrm{L}}$ measured using CPET) and that this fall in $\overset{.}{\mathrm{V}}{\mathrm{o}}_2$ at ${\widehat{\uptheta}}_{\mathrm{L}}$ would in turn be associated with increased harm (mortality at 1 year) following surgery.

In this manuscript, we describe the design of a prospective, observational, observer blinded cohort study investigating the effects of neoadjuvant cancer therapies (both chemo- and chemoradio-therapy – NAC/CRT) on exercise capacity and clinical outcome in patients undergoing surgery for UGI cancer.

### Aims

The aim of this study is to test the hypothesis that the decrease in physical fitness associated with NAC/CRT prior to UGI cancer resection may outweigh the benefits (duration of survival) achieved by NAC/CRT in some patients. Specifically, we will test the following hypotheses in this patient group:

Primary hypotheses:

1. Neoadjuvant cancer treatment will result in a decrease in physical fitness ($\overset{.}{\mathrm{V}}{\mathrm{o}}_2$ at ${\widehat{\uptheta}}_{\mathrm{L}}$), measured using CPET.
2. The change in physical fitness ($\overset{.}{\mathrm{V}}{\mathrm{o}}_2$ at ${\widehat{\uptheta}}_{\mathrm{L}}$) associated with neoadjuvant cancer treatment will be associated with mortality 1 year after surgery. This second hypothesis will be evaluated in two separate ways:
  A) The relative decrease in physical fitness ($\overset{.}{\mathrm{V}}{\mathrm{o}}_2$ at ${\widehat{\uptheta}}_{\mathrm{L}}$) associated with neoadjuvant cancer treatment prior to UGI cancer resection will be associated with mortality at 1 year after surgery.
    Secondary hypotheses:
  B) Patients whose physical fitness ($\overset{.}{\mathrm{V}}{\mathrm{o}}_2$ at ${\widehat{\uptheta}}_{\mathrm{L}}$) changes their risk stratification category (low risk $\overset{.}{\mathrm{V}}{\mathrm{o}}_2$ at ${\widehat{\uptheta}}_{\mathrm{L}}$ >14 ml.kg.^-1^min^-1^, medium risk $\overset{.}{\mathrm{V}}{\mathrm{o}}_2$ at ${\widehat{\uptheta}}_{\mathrm{L}}$ 11.0–14.0 ml.kg^-1^.min^-1^, high-risk $\overset{.}{\mathrm{V}}{\mathrm{o}}_2$ at ${\widehat{\uptheta}}_{\mathrm{L}}$ 8.0–10.9 ml.kg^-1^.min^-1^, highest risk $\overset{.}{\mathrm{V}}{\mathrm{o}}_2$ at ${\widehat{\uptheta}}_{\mathrm{L}}$ <8.0 ml.kg^-1^.min^-1^) following neoadjuvant cancer treatment would have an increased 1-year mortality following surgery when compared with those who do not increase risk stratification category.
3. Pre-NAC variables (including CPET derived variables) can be modelled to predict post NAC $\overset{.}{\mathrm{V}}{\mathrm{o}}_2$ at ${\widehat{\uptheta}}_{\mathrm{L}}$.
  Exploratory hypotheses:
4. The relative decrease in physical fitness ($\overset{.}{\mathrm{V}}{\mathrm{o}}_2$ at ${\widehat{\uptheta}}_{\mathrm{L}}$) associated with neoadjuvant cancer treatment prior to UGI cancer resection will be associated with i) increased post-operative morbidity, assessed using the Post-Operative Morbidity Survey (POMS), and ii) worse patient reported outcome assessed using EQ-5D quality of life questionnaire.
5. The relative decrease in peak oxygen consumption ($\overset{.}{\mathrm{V}}{\mathrm{o}}_2$ peak) associated with neoadjuvant cancer treatment prior to UGI cancer resection will be associated with i) increased post-operative morbidity, assessed using POMS, ii) worse patient reported outcome, assessed using EQ-5D quality of life questionnaire and iii) decreased 1 year survival.
6. Patients with a lower exercise capacity ($\overset{.}{\mathrm{V}}{\mathrm{o}}_2$ at ${\widehat{\uptheta}}_{\mathrm{L}}$ and $\overset{.}{\mathrm{V}}{\mathrm{o}}_2$ peak) tolerate NAC prior to upper gastrointestinal cancer resection (in terms of patient reported outcome and compliance with NAC protocol) less well than patients with a higher exercise capacity.
7. Patients who do not tolerate NAC prior to upper gastrointestinal cancer resection (in terms of PROMS and compliance with NAC protocol) have a worse postoperative outcome (1-year mortality, POMS, EQ-5D).
8. Changes in CPET derived variables will explain mechanisms of NAC associated exercise limitation.
9. CRT (CROSS style chemoradiotherapy) will result in a greater fall in $\overset{.}{\mathrm{V}}{\mathrm{o}}_2$ at ${\widehat{\uptheta}}_{\mathrm{L}}$ and $\overset{.}{\mathrm{V}}{\mathrm{o}}_2$ peak between pre- and post- neoadjuvant cancer treatments than NAC (MAGIC type chemotherapy).

## Methods

### Design

This study is planned as a multi-centre, prospective, observational, blinded (for physiological and surgical outcome assessments) observational cohort study, funded by the National Institute for Health Research for Patient Benefit Programme (PB-PG-0609-18262), approved for all NHS sites by the Dyfed Powys Research Ethics Committee (11/WA/0072) and registered with clinicaltrials.gov (NCT01325883). The study is to be conducted in four United Kingdom based NHS hospitals including; University Hospital Southampton NHS Foundation Trust, University Hospital Aintree NHS Foundation Trust, Lancashire Teaching Hospital and South Tees Hospital NHS Foundation Trust (above ethics number covers all four NHS sites).

### Participants

All patients listed to undergo NAC/CRT followed by elective UGI cancer resection (oesophagectomy and gastrectomy) are eligible for inclusion. Exclusion criteria are: inability to give informed consent, under 16 years of age, non-resectable disease, inability to perform CPET or bicycle exercise due to known contra-indication, and patients who declined surgery or neoadjuvant cancer treatment, or who received non-standard neoadjuvant cancer treatment.

### Recruitment

Patients who are candidates for curative surgery will undergo CPET both before and after neoadjuvant cancer treatment. All potentially eligible patients will be identified at the UGI multi-disciplinary meeting and approached with written information about the trial at the oncology/surgical outpatient appointment. All patients will be contacted by telephone to provide additional information about the trial and to confirm their eligibility. If the patient chooses to participate in the study, the first research visit is organised and written informed consent will be obtained together with all baseline measurements.

### Measurements

All patients will undergo a CPET and a patient reported outcome measure (PROM) questionnaire before neoadjuvant cancer treatment and also following completion of neoadjuvant cancer treatment (approximately 4 weeks following completion of neoadjuvant cancer treatment and immediately before planned surgery). Other outcome measures to be assessed following surgery are summarised in Table 1.

**Table 1:** Outcomes and assessment measures used in the study

Outcomes	Assessment measure	Pre-neoadjuvant cancer treatment	Post- neoadjuvant cancer treatment	Day 3 Post-surgery	Day 5 Post-surgery	Day 7 Post-surgery	Day 15 Post-surgery	Day 30 Post-Surgery	1-Year Post-Surgery
Primary endpoint									
Physical fitness	Cardiopulmonary exercise test (CPET)	X	X						
Secondary endpoint									
Physical fitness to assess risk stratification	Cardiopulmonary exercise test (CPET)	X	X						
Exploratory endpoint									
Post-operative morbidity	Post-Operative Morbidity Score (POMS)			X	X	X	X		
Patient Reported Outcome	EQ-5D	X	X					X	X
Survival									X

“X” denotes measurement obtained at that time point. CPET – Cardiopulmonary exercise test; POMS – Post-Operative morbidity Survey

### Chemotherapy vs. Chemoradiotherapy

Due to the advent of CRT treatment during the conduct of this study, an additional hypothesis (number 9 above) has been added to those proposed in the original study protocol to explore any differences in impact on physical fitness between neoadjuvant cancer treatments, namely NAC (MAGIC type chemotherapy) and CRT (CROSS style chemoradiotherapy).

### Primary outcome

#### Physical fitness ($\overset{.}{\mathrm{V}}{\mathrm{o}}_2$ at ${\widehat{\uptheta}}_{\mathrm{L}}$) derived using CPET

CPET will be used to assess physical fitness pre- and post-neoadjuvant cancer treatment. Patient and surgical characteristics recorded at first CPET will include age, gender, height, weight, diagnosis, staging, planned procedure. Height and weight will also be recorded at the second CPET. All CPET’s are performed in-hospital by trained and experienced staff with full resuscitation capability. All efforts will be made to conduct the tests to facilitate other clinical appointments. Each individual CPET is conducted at a similar time of day. Participants will be asked to refrain from caffeine ingestion and strenuous exercise prior to the test for at least 2 h. All CPET’s will be performed using an electromagnetically braked cycle ergometer (Ergoline 2000), 12 lead ECG, non-invasive blood pressure, pulse oximetry and a metabolic cart (Geratherm Respiratory GmbH, Love Medical Ltd). Patients were asked to perform an incremental ramp test to the limit of tolerance and to maintain a cycling cadence at 55–65 revolutions per minute (rmp) throughout the test. CPET assesses the amount of oxygen extracted from the inspired gas in a given period of time, expressed as $\overset{.}{\mathrm{V}}{\mathrm{o}}_2$ at ${\widehat{\uptheta}}_{\mathrm{L}}$ and $\overset{.}{\mathrm{V}}{\mathrm{o}}_2$ peak. Ventilatory equivalents for oxygen and carbon dioxide (${\overset{.}{\mathrm{V}}}_{\mathrm{E}}/\overset{.}{\mathrm{V}}{\mathrm{o}}_2$ and ${\overset{.}{\mathrm{V}}}_{\mathrm{E}}/\overset{.}{\mathrm{V}}{\mathrm{co}}_2$) are measurements of the ventilatory requirement for a given metabolic rate. Estimation of $\overset{.}{\mathrm{V}}{\mathrm{o}}_2$ at ${\widehat{\uptheta}}_{\mathrm{L}}$ will be performed using a conventional cluster of variables (breakpoint in the $\overset{.}{\mathrm{V}}{\mathrm{o}}_2$ and $\overset{.}{\mathrm{V}}{\mathrm{co}}_2$ relationship), with increases in ${\overset{.}{\mathrm{V}}}_{\mathrm{E}}/\overset{.}{\mathrm{V}}{\mathrm{o}}_2$ and end-tidal oxygen partial pressure but no increase in ${\overset{.}{\mathrm{V}}}_{\mathrm{E}}/\overset{.}{\mathrm{V}}{\mathrm{co}}_2$ or fall in end-tidal carbon dioxide partial pressure PETCO_2_ [8]. Evaluation will be undertaken independently by two experienced assessors, blinded to each other’s assessments, with disagreement resolved by a third assessor. The multi-disciplinary team caring for the patients will not be provided with any information regarding outcome measures.

#### Mortality at 1 year

Date of death will be obtained via the National Medical Information Service.

### Secondary outcomes

#### Physical fitness ($\overset{.}{\mathrm{V}}{\mathrm{o}}_2$ peak) and preoperative risk categories derived from CPET

CPET derived $\overset{.}{\mathrm{V}}{\mathrm{o}}_2$ at ${\widehat{\uptheta}}_{\mathrm{L}}$ will be to stratify patients into preoperative risk categories after neoadjuvant cancer treatment: low risk $\overset{.}{\mathrm{V}}{\mathrm{o}}_2$ at ${\widehat{\uptheta}}_{\mathrm{L}}$ >14.1 ml.kg^-1^.min^-1^; medium risk $\overset{.}{\mathrm{V}}{\mathrm{o}}_2$ at ${\widehat{\uptheta}}_{\mathrm{L}}$ 11.0–14.0 ml.kg^-1^.min^-1^; high-risk $\overset{.}{\mathrm{V}}{\mathrm{o}}_2$ at ${\widehat{\uptheta}}_{\mathrm{L}}$ 8.1–10.9 ml.kg^-1^.min^-1^; highest risk $\overset{.}{\mathrm{V}}{\mathrm{o}}_2$ at ${\widehat{\uptheta}}_{\mathrm{L}}$ <8.0 ml.kg^-1^.min^-1^. CPET derived $\overset{.}{\mathrm{V}}{\mathrm{o}}_2$ peak will be defined as the average $\overset{.}{\mathrm{V}}{\mathrm{o}}_2$ over the last 30 s of exercise.

#### Postoperative morbidity

Post-operative morbidity will be objectively recorded using POMS at day 3, 5, 7 and 15 days following surgery, in order to explore the relationship between neoadjuvant cancer treatments, surgery and short-term post-operative harm. The POMS [9, 10] is a validated measure of short-term post-operative harm which includes an 18-item tool that addresses nine domains of morbidity relevant to the post-surgical patient: pulmonary, infection, renal, gastrointestinal, cardiovascular, neurological, wound complications, haematological and pain. For each domain either presence or absence of morbidity will be recorded on the basis of precisely defined clinical criteria.

#### Patient reported outcomes measure

Patient Reported Outcomes will be described using EQ-5D-5 L [11], which has been recommended for use as a generic PROM following major surgery. This will be measured at several time points; pre- and post-neoadjuvant cancer treatment, and 30-days and 1-year post-surgery. This questionnaire includes a health scale and also encompasses the following 5 domains; 1) mobility, 2) self-care, 3) usual activities, 4) pain/discomfort 5) anxiety/depression.

### Data elaboration and statistical analysis

#### Sample size calculation

Hypothesis 1: A sample of 152 patients would be required to detect a difference in 1.0 ml.kg^.-1^.min^-1^ of $\overset{.}{\mathrm{V}}{\mathrm{o}}_2$ at ${\widehat{\uptheta}}_{\mathrm{L}}$ using a paired t-test at the 5 % significance level with 90 % power. This is assuming the standard deviation of the difference in $\overset{.}{\mathrm{V}}{\mathrm{o}}_2$ at ${\widehat{\uptheta}}_{\mathrm{L}}$ values is 3.8 ml.kg^.-1^.min^-1^. A smaller sample of 114 patients would provide 80 % power.

Hypothesis 2A: A sample size of 242 is required to detect a difference in 1 year mortality rates of 15 % [30 % versus 15 %] between the two AT change groups [no change/deteriorate] using a chi-squared test at the 5 % significance level with 80 % power, assuming equal numbers of patients in both groups. A smaller sample of 104 patients will be able to detect a difference of 23 % [34 % vs 11 %] with 80 % power.

The aim for hypothesis 2A is to compare the predictive ability of both models to ascertain how prognostic the relative decrease in exercise capacity is, after adjusting for baseline exercise. Approximately 250 patients are required to develop reliable prediction models containing 5 factors [e.g. age, gender, centre, location of tumour, laparoscopic vs. open]. This is using the “Rule of 10” and assumes that the 1-year mortality rate is 20 % [conservative estimate]. Given a 30 % non-completion of NAC and 2 CPET tests based on Liverpool data, approximately 360 patients will need to be recruited. With a smaller sample of 104 patients and 25 % mortality rate, we can develop reliable prediction models (using standard methods) that contain fewer factors, e.g. 3. In addition, we plan to use “penalised” regression models that are able to contain more factors, even when the “Rule of 10” is not met [12].

We therefore set out to recruit 250 patients into this study in 4 centres over 24 months. The volume of eligible patients in these 4 centers is approximately 200 patients per year. We therefore estimate that the study recruitment time would take less than 24 months based on a 66 % recruitment rate (132 patients per year).

#### Procedures for data monitoring and entry

Data will be inputted by double-data entry and data validation will take place according to the procedures set out in the data management plan and data validation plan. Prior to any statistical analysis, all variables will be checked for number of missing values, impossible and improbable values. Impossible and improbable values will be defined by clinical opinion. Improbable values will also include values that are outside three standard deviations of the mean value, any questions regarding the data will go back to the data manger. Descriptive statistics will be calculated for all variables and distributional assumptions checked.

#### Statistical analysis

Descriptive analyses will be carried out to summarise patient characteristics. Continuous variables will be presented as either mean (standard deviation) or median (inter-quartile) as appropriate. The distribution of the continuous variables will be investigated using histograms. Categorical variables will be presented as frequency (%).

The primary analysis will be a comparison of physical fitness ($\overset{.}{\mathrm{V}}{\mathrm{o}}_2$ at ${\widehat{\uptheta}}_{\mathrm{L}}$) before and after neoadjuvant cancer treatment using a paired t-test. Distributional assumptions will be assessed using a normal plot. If these are not met then Wilcoxon matched-pairs signed-ranks test will be used. Survival at 1 year will be compared between ‘change in fitness’ groups using either the χ2 test (or Fisher’s exact test if cell counts are insufficient) or log-rank test depending on the degree of censoring throughout the year. Either logistic or Cox regression methods will be used to investigate the relationship between fitness and mortality, adjusted for confounders. The calibration and discrimination of these models will be assessed. The relationship between morbidity and fitness will be explored using mixed models that account for the fact that morbidity is measured on several occasions post-surgery.

All estimates will be given with 95 % confidence intervals. Missing data will be investigated though imputation is not planned as we do not expect a large proportion of missing data. All analyses will be performed with the statistical software Stata 14.0.

## Discussion

Oesophageal and gastric neoadjuvant cancer treatments together with surgery have been associated with 1-year mortality [7]. The reliability and association of CPET variables with outcome following major surgery has now been established [13–15]. Selected CPET variables like the $\overset{.}{\mathrm{V}}{\mathrm{o}}_2$ at ${\widehat{\uptheta}}_{\mathrm{L}}$ and $\overset{.}{\mathrm{V}}{\mathrm{o}}_2$ peak have the utility of identifying high-risk surgical patients, prior to a variety of major surgical procedures. Additionally, other variables derived from CPET such as the ventilatory equivalent ratio for carbon dioxide (${\overset{.}{\mathrm{V}}}_{\mathrm{E}}/\overset{.}{\mathrm{V}}{\mathrm{co}}_2$) are associated with post-operative outcome in several surgical patient groups [16–19].

At present there is little evidence supporting the use of pre-operative CPET to aid patient’s and clinician’s decisions in relation to surgical risk, especially pertaining to oesophago-gastric surgery. Furthermore, evidence supporting pre- and post- cancer therapy CPET prior to major surgery as a useful risk-stratification and prognostic tool is also very limited. Assessing physical fitness before neoadjuvant cancer treatment may provide additional information that allows clearer risk-assessment and risk-mitigation by perioperative physicians. This will also better assist cancer patients in their decision-making process and consent. Identification of factors predictive of the response to neoadjuvant cancer treatment would allow better targeting of cancer treatment and improve the quality of information informing both the multi-disciplinary team and patient decisions.

The findings from this study are likely to be of particular importance in patients with borderline initial fitness where further loss of fitness after neoadjuvant cancer treatment may be critical, and in whom over-all survival may even be improved by proceeding directly to surgery. The study aims to answer important clinical questions including;

1. Is neoadjuvant cancer treatment (NAC/CRT) associated with a reduction in physical fitness before surgery?
2. In less fit patients, is this reduction related to worse clinical outcomes (mortality and morbidity)?
3. Is NAC/ CRT associated with a reduction in quality of life following cancer treatment and before surgery?
4. Is there an association suggesting that it might be possible to select patients for NAC/ CRT based on physical fitness refined using objectively measured CPET variables?

Strengths of this study include the use of blinded data collection and analysis, double data entry and the robust statistical analyses employed. Furthermore, other strengths of this study include the homogeneous study population, the clearly defined inclusion and exclusion criteria, the number of contributing centres, the wide ranging geographical distribution (in an attempt to reduce bias due to socioeconomic status and geography), the robust reporting of objectively measured CPET variables, and the use of the POMS as a primary outcome measure for morbidity. Additionally, reporting of CPET safety aspects, medium term survival follow-up, blinding of the data analyses and the double data entry further reinforce the study design.

## Conclusion

The results of this study will inform testing of the above mentioned hypothesis which might allow individualisation of the treatment pathway for patients with locally advanced oesophago-gastric cancers. That might mean that patients who are unfit have a survival advantage after surgery if they do not have NAC/CRT. Furthermore, patients would have a tailored risk-stratification prior to major surgery and therefore would be better informed prior to consenting for cancer major surgery. The principal benefits of this study, if the underlying hypothesis is correct, will be improved information to clinicians allowing better patient selection for neoadjuvant cancer therapies and major surgery, together with informing larger clinical trials aiming to improve clinical outcomes (reduced mortality and morbidity). Finally, our group is exploring the effects of tailored preoperative exercise intervention strategies during and after neoadjuvant cancer treatments in this patient group to assess whether increasing preoperative physical fitness may improve postoperative outcome. Tailoring the correct treatment plan to the correct patient may therefore increase survival. Results of the current study will be available at the end of 2016.

## Abbreviations

OG: Oesophago-gastric
NAC: Neoadjuvant chemotherapy
CPET: Cardiopulmonary exercise testing
CRT: Chemoradiotherapy
UGI: Upper Gastrointestinal
$\overset{.}{\mathrm{V}}{\mathrm{o}}_2$ at ${\widehat{\uptheta}}_{\mathrm{L}}$: Oxygen uptake at estimated lactate threshold
$\overset{.}{\mathrm{V}}{\mathrm{o}}_2$ peak: Peak exercise
POMS: Post-operative morbidity survey
NHS: National health service
PROM: Patient reported outcome measure
RMP: Revolutions per minute
${\overset{.}{\mathrm{V}}}_{\mathrm{E}}/\overset{.}{\mathrm{V}}{\mathrm{o}}_2$: Ventilatory equivalents for oxygen
${\overset{.}{\mathrm{V}}}_{\mathrm{E}}/\overset{.}{\mathrm{V}}{\mathrm{co}}_2$: Ventilatory equivalents for carbon dioxide
PETCO_2_: End-tidal carbon dioxide partial pressure
SD: Standard deviation
IQR: Inter-quartile range
95 % CIs: 95 % confidence intervals
